# Investigation of a memory effect in a Au/(Ti–Cu)Ox-gradient thin film/TiAlV structure

**DOI:** 10.3762/bjnano.13.21

**Published:** 2022-02-24

**Authors:** Damian Wojcieszak, Jarosław Domaradzki, Michał Mazur, Tomasz Kotwica, Danuta Kaczmarek

**Affiliations:** 1 Wrocław University of Science and Technology, Faculty of Electronics, Photonics and Microsystems, Janiszewskiego 11/17, 50-372 Wroclaw, Poland

**Keywords:** gradient thin film, magnetron sputtering, memory effect, resistive switching

## Abstract

This paper presents the results of the analysis of resistive switching properties observed in a Au/(Ti–Cu)Ox/TiAlV structure with a gradient distribution of Cu and Ti along the (Ti–Cu)Ox thin film thickness. Thin films were prepared via multisource reactive magnetron co-sputtering. The programmed profile of the pulse width modulation coefficient during sputtering of the Cu target allowed us to obtain the designed gradient U-shape profile of the Cu concentration in the deposited thin film. Electrical measurements of the Au/(Ti–Cu)Ox/TiAlV structure showed the presence of nonpinched hysteresis loops in the voltage–current plane testifying a resistive switching behavior. Results of optical, X-ray, and ultraviolet photoelectron spectroscopy measurements allowed us to elaborate the scheme of the bandgap alignment of the prepared thin films with respect to the Au and TiAlV electrical contacts. Detailed structure and elemental profile investigations allowed us to conclude about the possible mechanism for the observed resistive switching mechanism.

## Introduction

In recent years, significant development has been observed in design, simulation, manufacturing, and characterization of devices with the ability to switch between two resistance states, namely low-resistance (LRS) and high-resistance (HRS) states, the so-called resistive switching devices. This increase is due to the possible application of such devices in the fields of neuromorphic [1–3] and chaotic systems [4–5], textile electronics [6], and even quantum systems [7]. Resistive switching devices have already found their place in the field of memory applications, especially in non-volatile memory such as resistive random access memory (RRAM) [8–12] or conducting bridge random access memory (CBRAM) [13]. Resistive switching devices are usually made in the form of a metal–insulator–metal (MIM) structure [14]. Commonly, such structures are fabricated in the form of a stack of multilayers consisting of a very thin (usually several nanometers) layer of insulating oxide and a much wider (several hundred nanometers) semiconducting (e.g., doped or nonstoichiometric oxide) layer. Materials used either for insulating or semiconducting layers include HfO_2_ [3,15–18], ZnO [19–20], CuO [21–27], ZrO_2_ [12], Ta_2_O_5_ [28–29], and NiO [3,30–32]. The most commonly used material in resistive switching devices is TiO_2−_*_x_* [33–38]. In addition to the oxide layers, the material used for metal electrodes plays another crucial role in the resistive switching mechanism. Usually, materials such as Au, Ag, Ni, Ti, W, TiN, or ITO are used [2,21,39]. Some examples of resistive switching behavior were also found in structures based on nanowires [40] or nanotubes [25,40], where the resistive switching device is characterized by the presence of a pinched or nonpinched hysteresis loop in the *I*–*V* characteristics in the DC plane.

Our previous works [41–42] have shown that an interesting alternative to the well-known conventional structures, with a multilayer stack of thin films with different electrical conducting and nonconducting properties described above, could be a thin film the composition of which changes along its thickness. So far, a memristive-like memory effect has been observed for thin film structures with a so-called V-type [41] or linear [42] gradient profile of the Cu distribution in (Ti–Cu)Ox thin films. In the present paper, the results for (Ti–Cu) oxide semiconducting thin films prepared with a U-shape distribution profile of copper are presented. Experimental electrical measurements performed using DC simulation showed wide nonpinched hysteresis loops, indicating bipolar resistive switching properties. Additionally, the so-called forming process of switching properties has been observed for the first cycle of the current-to-voltage measurements. On the basis of the performed investigations, we suggest that the conducting filament switching is the most probable mechanism. This is supported by investigations of structure and surface properties using X-ray methods, UV photoelectron spectroscopy, and cross-sectional elemental analysis.

## Experimental

The deposition system and the method for the preparation of gradient thin films have already been described in detail in [41–45]. The thin films were deposited via reactive magnetron co-sputtering, using two circular titanium targets (99.995%) and one circular copper target (99.995%). All three targets were sputtered simultaneously in an oxygen atmosphere of 99.999% purity. The targets used in the process were 28.5 mm in diameter and their thickness was 3 mm. The process uses magnetrons with an unbalanced magnetic field set in a confocal configuration vs the substrate. The target–substrate distance was 14 cm. The unbalanced magnetic configuration system was applied. Before the deposition process, the working chamber was pumped to a base pressure of 10^−3^ Pa. Thin films were sputtered without additional intentional heating of the substrates during the process. It can be assumed that the substrate temperature did not exceed a temperature of 373 K. Each magnetron was powered with a separate MSS2 power supply from Dora Power System. The applied power supply allowed to obtain a maximum power of up to 2 kW in the unipolar pulsed DC mode. The power delivered to the magnetrons was independently controlled for each of the magnetrons [43]. DC pulses of the magnetron power supply consisted of groups of unipolar sinusoidal pulses with a frequency of 140 kHz, whereas the power supplied to the magnetrons was regulated by changing the width of the groups of these pulses (pulse width modulation, PWM). One of the biggest difficulties in the preparation of mixed (Ti,Cu)Ox thin films with defined composition using co-sputtering of Ti and Cu targets is that titanium and copper differ from each other in the sputtering yield in oxygen by a factor of more than ten. Therefore, to increase the sputtering flux of titanium species, two Ti and one Cu targets were sputtered in the so-called simultaneous mode. The magnetrons were arranged in a confocal configuration. To obtain a gradient distribution of elements as a function of the thickness of the deposited layers, magnetrons equipped with titanium targets were supplied with a constant coefficient pwm_Ti_ = 100% throughout the deposition process. On the contrary, the magnetron with the cooper target was powered with a pwm_Cu_ coefficient the value of which was changed from 60% to 10% and 0% for the half of the deposition time, and then for the remaining half of the deposition time, it was changed from 0% to 10% and to 60% (see Figure 8 below). The total time of deposition was 240 min. The determined values of the pwm_Ti_ and pwm_Cu_ coefficients were selected on the basis of preliminary deposition processes.

Due to the requirements of the different characterization methods, thin gradient layers were deposited on silicon (Si), amorphous silica (SiO_2_) and conductive metallic substrates (Ti6Al4V). The resulting thickness of the prepared thin films was about 610 nm as measured using a Talysurf optical profiler (Tylor Hobson CCI Lite). Additionally, circular 1 mm gold pads were evaporated on top of the prepared structure to allow for electrical characterization. The average material composition of the gradient thin film was determined using X-ray microanalysis employing an EDAX Genesis energy-dispersive spectrometer (EDS) as part of the FESEM FEI Nova NanoSEM 230 scanning electron microscope. The content of Ti and Cu in the entire volume of the thin film was estimated to be 48 atom % and 52 atom %, respectively. Additionally, the material composition was determined without taking oxygen into account, and no unintentional impurities were observed in the coating. Structural properties were analyzed using X-ray diffraction (XRD) and transmission electron microscopy (TEM). XRD patterns were obtained with a PANalytical Empyrean PIXed3D powder diffractometer with Cu Kα radiation (1.5406 Å) and no diffraction peaks were observed testifying the predominantly amorphous nature of the deposited thin films. The microstructure of the (Ti–Cu)Ox films was further analyzed with the aid of a TECNAI G2 FEG Super-Twin (200 kV) transmission electron microscope equipped with EDS attachment. The local chemical composition of the cross section was also investigated to show the gradient distribution of Ti and Cu as a function of the thin film depth. X-ray photoelectron spectroscopy (XPS) studies were performed to determine the chemical state of titanium and copper on the surface of the mixed oxide thin films. A Specs XR-50 X-ray non-monochromatic excitation source with Mg Kα radiation (1253.6 eV) was used. A Specs Phoibos 100 MCD-5 (5 single-channel electron multiplier) hemispherical analyzer was used to collect photoelectrons with a step size of 0.1 eV. All spectra were calibrated with respect to the binding energy of the adventitious C 1s peak at 284.8 eV. Ultraviolet photoelectron spectroscopy (UPS) was performed using a non-monochromatic He I line (21.22 eV) excitation source with a step size of 0.025 eV. A bias voltage of −5 V was applied to the thin film sample during UPS measurements to obtain a clear secondary electron cutoff. The binding energies of the spectra were referred to the Fermi level (*E*_F_) determined from a cleaned reference Au sample. Measurement results were analyzed with the aid of CasaXPS software. In the case of XPS and UPS measurements, the results were averaged over a certain surface area, that is, the beam diameter was approximately 5 nm. To determine the type of electrical conductivity, thermoelectrical Seebeck effect measurements were conducted using a setup consisting of an INSTEC chamber equipped with four electrical probes and two hot chucks, an INSTEC MK1000 temperature controller, and an INSTEC LN2-P pump. The optical transmission coefficient in the visible part of the optical spectrum was measured using a scientific grade CCD QE65000 spectrophotometer (Ocean Optics). For DC current-to-voltage electrical measurements, a Keithley SCS4200 semiconductor characterization system and a M100 Cascade Microtech probe station were used. All electrical measurements were made at controlled room temperature (23 °C) and humidity (30% RH) in ambient air.

## Results

### Electrical properties

**Figure 1:**
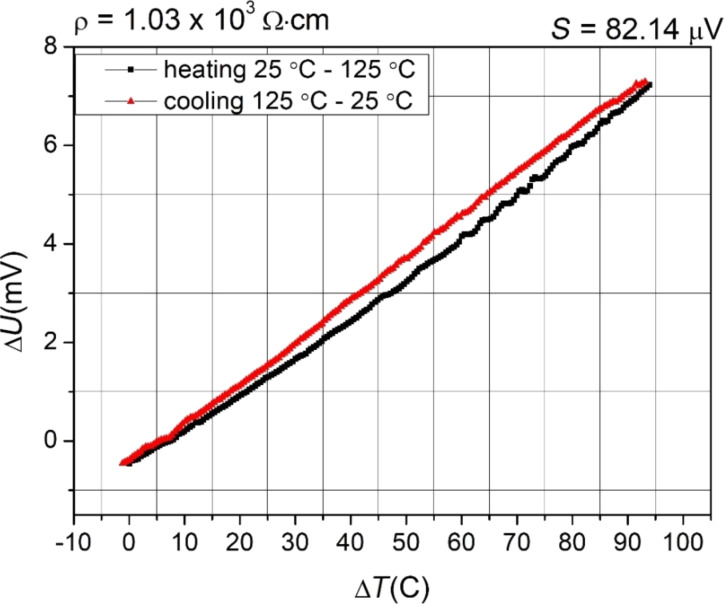
Characteristics of the thermoelectrical voltage of a (Ti–Cu)Ox thin film.

The resistivity of the thin film was determined to be 1 × 10^3^ Ω·cm. The type of electrical conductivity was determined on the basis of the sign of the thermoelectrical voltage. The Seebeck coefficient measurements were made in the range from 25 to 125 °C. The thermoelectrical voltage as a function of the temperature difference measured between two opposite electrical contacts is shown in Figure 1.

The Seebeck coefficient (+82.14 μV) testified the p-type conductivity of the prepared gradient thin film. The p-type of electrical conduction is often reported for Cu_2_O (or CuO)-based thin films, while TiO_2_ is an n-type oxide [46–47]. In the case of the prepared mixed (Ti–Cu)Ox thin film, the result obtained clearly testifies that holes are the major charge carriers.

**Figure 2:**
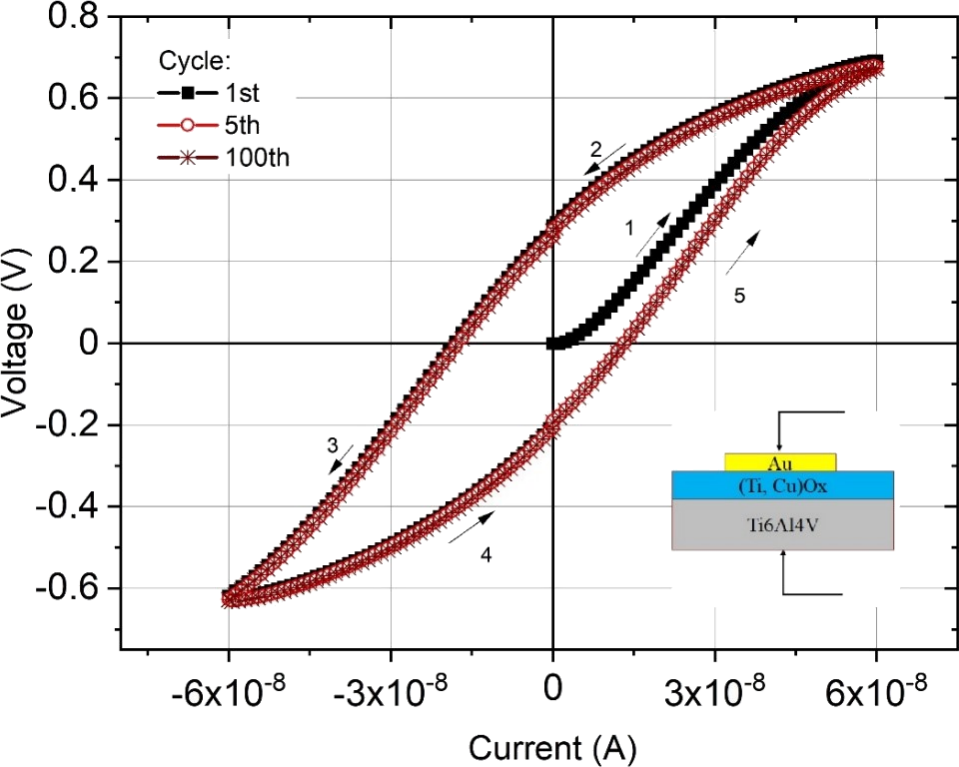
Direct current-to-voltage characteristics of the Au/(Ti–Cu)Ox/TiAlV thin film structure. Arrows indicate the sequence of changes in current forcing during measurements.

DC measurements of the current-to-voltage characteristic were performed in a transverse Au/(Ti–Cu)Ox/Ti6Al4V configuration system (Figure 2). The structure was powered by forcing a constant current, which was swept from 0 to 60 nA, then 60 nA → 0 nA → −60 nA and again −60 nA → 0 nA → 60 nA. Depending on the direction of the applied current, the structure was either in a high-resistance state (HRS) or in a low-resistance state (LRS) at the same absolute value of the current. When the direction of the current was changed to the opposite polarity, the structure still “remembered” its high-resistance state until, again, the direction of the current was changed and the structure returned to its low-resistance state. Measurements of the *I*–*V* characteristics were performed for several tens of cycles.

**Figure 3:**
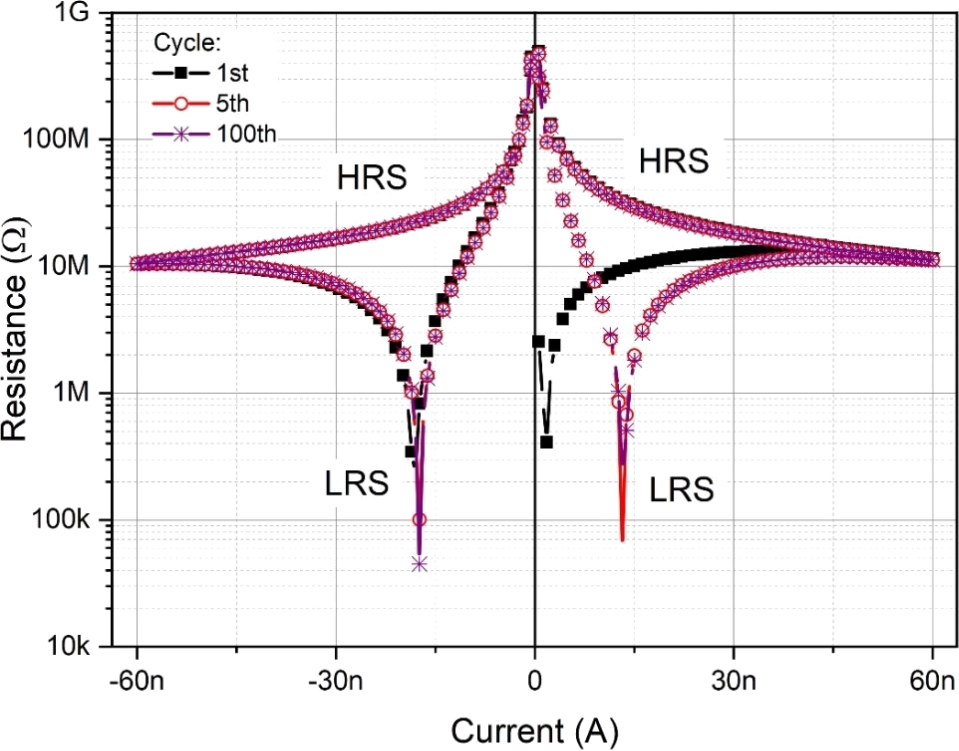
Switching characteristics for the Au/(Ti–Cu)Ox/Ti6Al4V thin film structure as a function of the forcing current for both directions of the current flow.

LRS operation was reached when the forcing current was about 1.32 × 10^−8^ A (or −1.74 × 10^−8^ A) resulting in a structure resistance of about 50 kΩ. However, for the same value of forcing current in the HRS, the structure resistance reached about 30 MΩ. Resistance-to-forcing current characteristics with observed switching behavior are presented in Figure 3.

**Figure 4:**
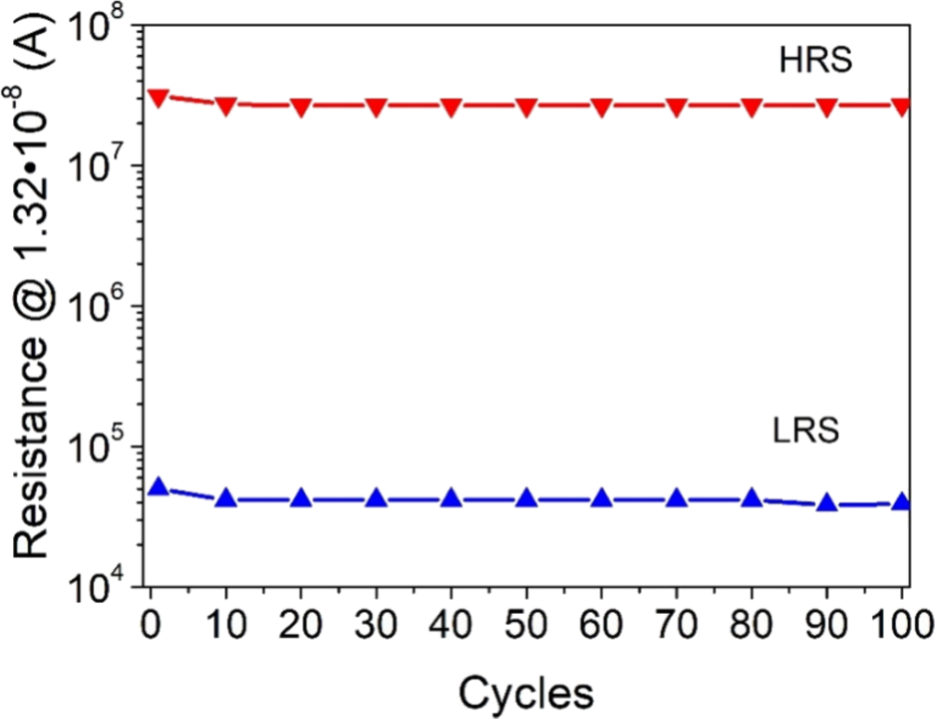
Retention characteristics for the Au/(Ti–Cu)Ox/TiAlV thin film structure.

The results obtained from the 100 measured cycles testify a very good reproducibility and good stability (retention) of the prepared material (Figure 4). The ratio of the structure resistance between the HR and LR states was approximately 6 × 10^2^.

### Optical properties

**Figure 5:**
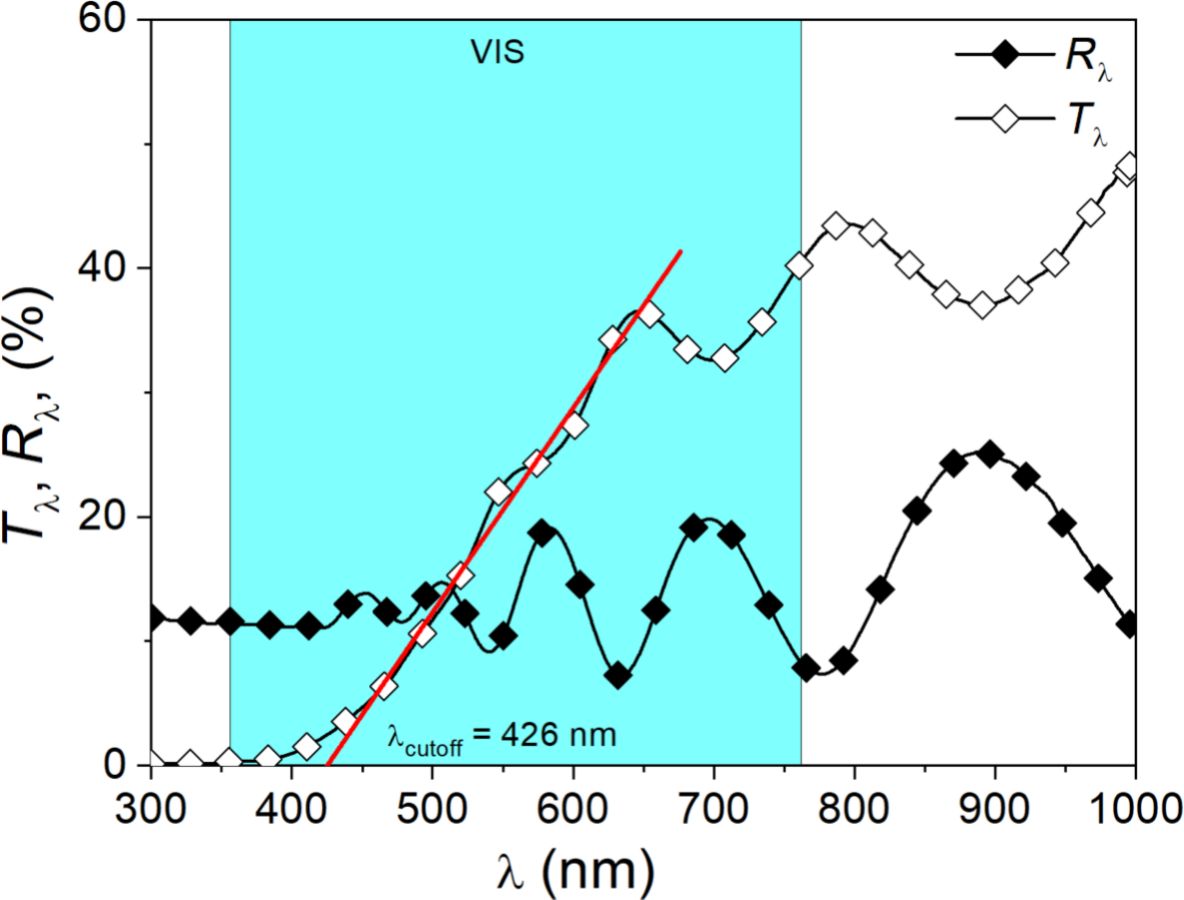
Transmission and reflection characteristics for gradient (Ti–Cu)Ox thin film.

Figure 5 presents the transmission and reflection spectra of the prepared thin (Ti–Cu)Ox film. As one can see, the prepared thin film is quite transparent in the visible part of the optical spectrum. However, the average transmission does not exceed more than 25% on average in the wavelength range of 500 to 1000 nm. In the infrared, the transparency of the thin film is higher and reaches 40% on average. Visible maxima and minima result from multiple interferences of the light reflected from interfaces between air and thin film and thin film and SiO_2_ substrate. From the optical spectra, an optical bandgap width of about 2.8 eV was determined for the allowed indirect transitions using the Tauc method.

### Structure and elemental composition

#### Surface properties

**Figure 6:**
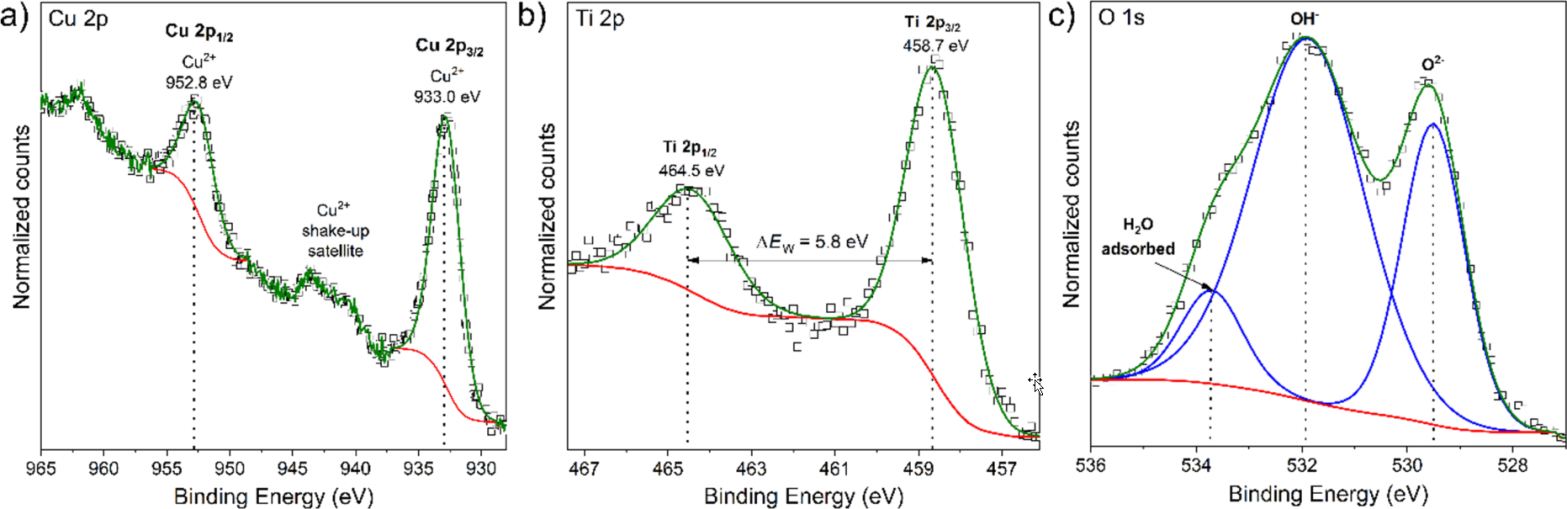
XPS spectra of the surface of (Ti_0.48_Cu_0.52_)Ox thin film: a) Cu 2p, b) Ti 2p, and c) O 1s core levels.

The oxidation state of copper on the surface of (Ti_0.48_Cu_0.52_)Ox thin film was analyzed with the XPS Cu 2p core level spectrum (Figure 6). The Cu 2p core level has split spin–orbit components with ΔBE of 19.8 eV and an intensity ratio of Cu 2p_1/2_ and Cu 2p_3/2_ of approximately 0.5. It is possible to distinguish Cu oxidation states taking into consideration not only the position of the Cu 2p_3/2_ peak but also the satellite features that could be visible above the binding energy of this peak. According to Biesinger [48–49], the shake-up satellite peaks are present for samples containing Cu^2+^ species but are absent for samples containing only Cu^0^ and Cu^+^ species. Therefore, in the case of the measured thin film, the binding energy of Cu 2p_3/2_ and the occurrence of well-visible satellite peaks at ca. 940–945 eV indicate the presence of Cu^2+^ species related to the CuO oxide [48–50].

The XPS spectrum of the Ti 2p core level is presented in Figure 6b. The position of the Ti 2p doublet and the binding energy separation between Ti 2p_3/2_ and Ti 2p_1/2_ (marked in the figure as Δ*E*_W_) equal to 5.8 eV testifies the oxidation state +4 of titanium present at the surface. The ratio between the areas of the Ti 2p_3/2_ and Ti 2p_1/2_ peaks is equal to 2:1, which confirms the presence of stoichiometric TiO_2_ at the surface of (Ti_0.48_Cu_0.52_)Ox thin film. Furthermore, the O 1s spectrum (Figure 6c) was deconvoluted into three peaks related to lattice oxygen (for TiO_2_ and CuO), hydroxy groups (OH^−^) and adsorbed water molecules (H_2_O_ads_).

**Figure 7:**
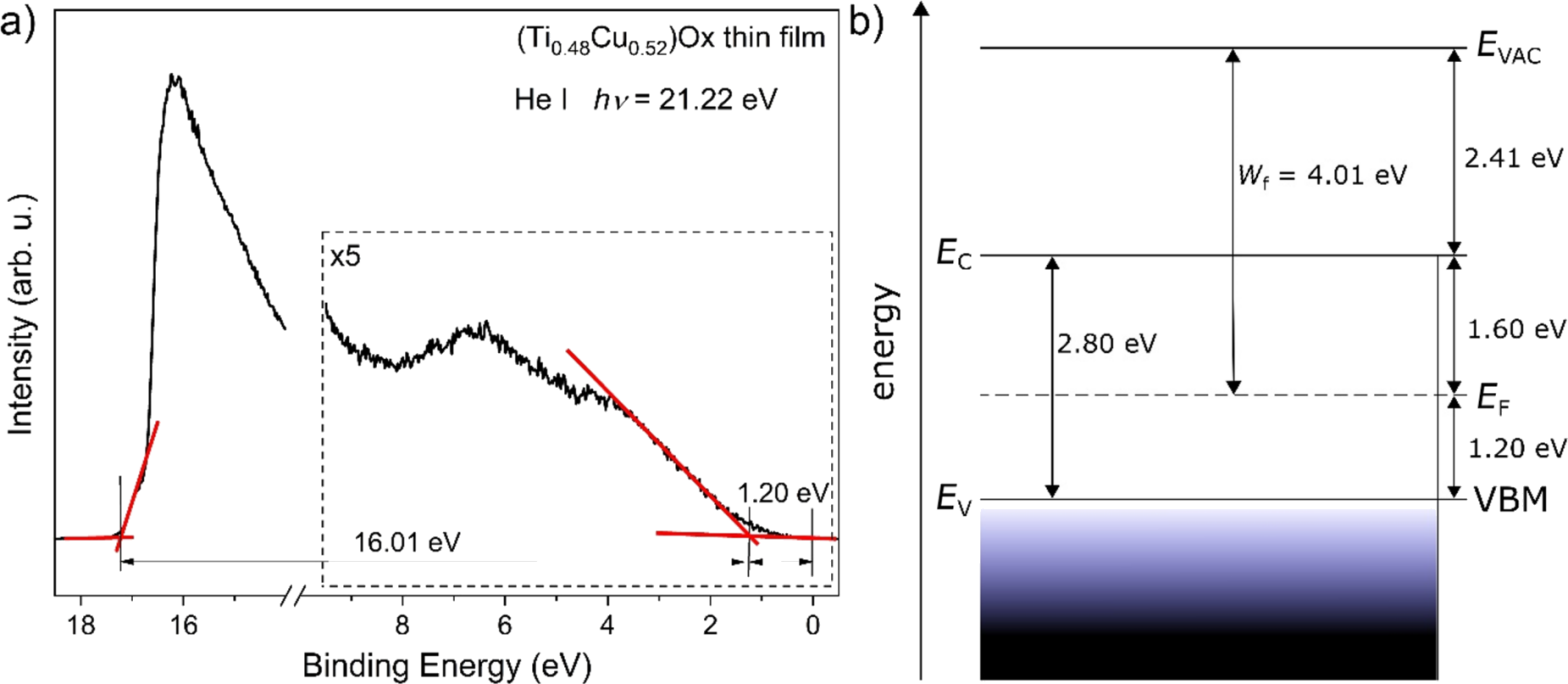
(a) Photoelectron spectrum of the valence band, (b) schematic energy diagram of the surface of the (Ti_0.48_Cu_0.52_)Ox thin film.

The UPS spectrum of the (Ti_0.48_Cu_0.52_)Ox thin film is shown in Figure 7. The position of the valence band maximum (VBM) was determined from the extrapolation of the line fit to the leading edge of the spectrum as marked in Figure 7a; it is 1.20 eV below the Fermi level (*E*_F_). Taking into consideration the bandgap energy of the thin films equal to 2.80 eV, the thin film surface exhibits p-type conduction. The electron affinity (χ) of the thin film surface was equal to 2.41 eV and was calculated based on the relationship [51–52]:


[1] $$\chi =h\nu -W-E_{\text{g}},$$

where *h*ν = 21.22 eV is the He (I) photon energy, *W* = 16.01 eV is the width of the spectrum, that is, the energy difference between the VBM and the photoemission cutoff energy, and *E*_g_ = 2.80 eV is the bandgap energy calculated from the transmission spectrum. The work function (*W*_f_) was determined to be equal to 4.01 eV and was calculated as the difference between the photon energy of the He (I) line and the position of the cutoff energy of the photoemission (17.21 eV).

#### Cross-sectional analysis

**Figure 8:**
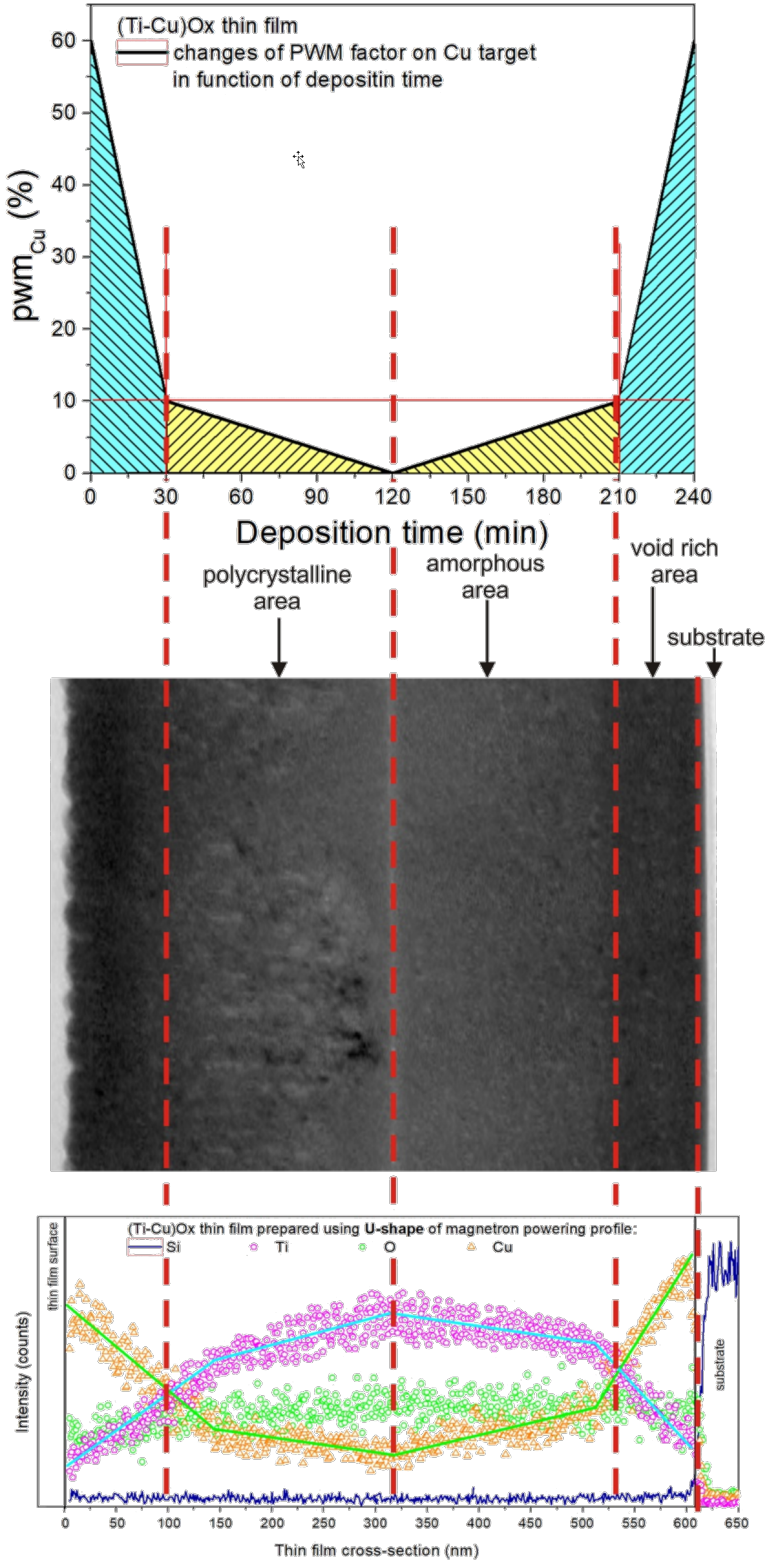
Results of TEM analysis and distribution of Cu, Ti, O, and Si in the gradient (Ti–Cu)Ox thin film with correlation to U-shaped-like changes of the pwm coefficient.

The structural investigations included measurements of the material composition using a scanning electron microscope (FEI Inspected S50) with an electron dispersion spectrometer (EDS) and the cross-sectional analysis of the prepared thin film structures using a transmission electron microscope (TEM) with X-ray probe. With respect to the programmed U-shape of the magnetron powering profile, the distribution of elements along the thickness of the deposited (Ti–Cu)Ox thin films consist of four parts. The first two parts include the decrease of copper in a more rapid way at the beginning of thin film deposition to ca. 80 nm and in a moderate way for the next ca. 200 nm of the thin film thickness. After that, the content of copper in the thin film structure begins to increase. The third part of the structure includes the point at ca. 350 nm where the content of copper is the lowest, while the content of titanium is the highest. After that point, the copper content starts to increase for the next ca. 200 nm. The fourth and last part is the rapid increase in copper content for the next ca. 80 nm. TEM and EDS (Figure 8) confirmed that a symmetric U-shaped gradient (Ti–Cu)Ox of the thin film was achieved. In addition, the structure of the prepared thin film could also be divided into three areas: (1) a polycrystalline area located from the surface to the near center of the structure, (2) an amorphous area located from the center to the near-substrate region, and (3) a void-rich area located from the near-substrate to the substrate region. In the middle of the structure there is a very thin (ca. 10 nm) region with different structural properties and chemical composition. From the performed TEM analysis (Figure 8) it can be interpreted as an amorphous area. During the deposition process, the flow of oxygen supplied to the working chamber was constant. Decreasing the power supplied to the magnetron with the copper target to 0% pwm results in the fact that all oxygen in the chamber was consumed by the sputtered titanium targets. This resulted in the deposition of dense amorphous TiO_2_ just in the middle of the gradient thin film.

## Discussion

**Figure 9:**
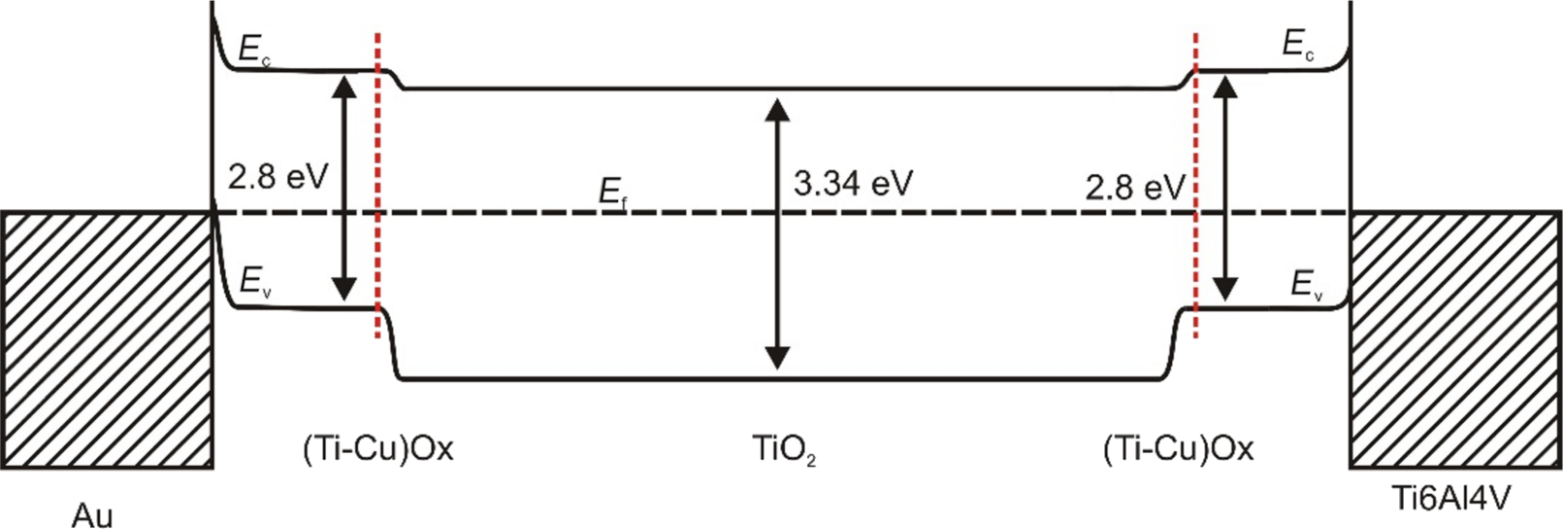
Schematic illustration of energy level diagrams of the prepared Au/(Ti–Cu)Ox/Ti6Al4V structure. The change in material composition in the film is marked by red lines.

According to the construction, structure, and elemental analyses, four different boundaries can be recognized in the prepared thin film structure: Au/(Ti–Cu)Ox, (Ti–Cu)Ox/TiO_2_, TiO_2_/(Ti–Cu)Ox, and (Ti–Cu)Ox/Ti6Al4V. To further analyze the switching mechanism of the observed memory effect, the energy diagram in Figure 9 was proposed. The constructed diagram assumes the presence of an additional wide TiO_2_ region in the middle of the prepared structure that corresponds to the region of high Ti concentration in the central part of the prepared (Ti–Cu)Ox thin film. The value of the work function (5.4 eV) and the width of the optical energy gap (3.34 eV) for TiO_2_ were determined for a reference (about 100 nm thick) amorphous TiO_2_ layer, prepared in the magnetron sputtering process using the same deposition system with the coefficient pwm_Ti_ = 100% during the entire sputtering process. The analysis allowed us to conclude that both interfaces created on the border of Au or Ti6Al4V and the deposited (Ti–Cu)Ox thin film were electrical ohmic contacts, because the value of the work function of Au (ΦAu = 5.3 eV) or (ΦTi6Al4V = 4.3 eV) is greater than the value of the work function of the layer (Φ(Ti–Cu)Ox = 4.01 eV). This conclusion is supported by the symmetric shape of the *I*–*V* curves measured in both directions of the forcing current (Figure 2).

Our previous works [41–42] have shown that structures based on (Ti–Cu)Ox thin films with either linear or V-type gradient profile of the Cu distribution along the thickness of the thin film resulted in a pinched current-to-voltage behavior indicating a memristive-like memory effect. In the present case, the measured *I*–*V* curves showed a nonpinched hysteresis shape. We believe that this effect could be due to the wide (about 420 nm) area of the middle region, rich in titanium oxide, which resulted in a relatively high series resistance. In the discussed case, switching from the high- to the low-resistance state could be connected with the formation of filament-type conduction paths that allow electrical charge carriers to flow between two opposite contacts. Conductive filaments are often reported in the literature as a mechanism responsible for the resistive switching behavior occurring in conventional multilayer stack constructions. Conducting paths are usually formed over extended defects in the thin film structure as a result of a thermal mechanism. An important property of this effect is the occurrence of the forming process, which occurs when the structure under test is first stimulated by an electric current (or voltage) in a cyclic *I*–*V* measurement.

## Conclusion

The paper presents the results of investigations of the memory effect observed in a thin-film structure with a U-shape gradient profile of the Cu distribution prepared using magnetron co-sputtering. Structural, elemental, and bandgap analyses allowed for a discussion of the observed switching effect of memory testified by the measured nonpinched *I*–*V* curves. It was concluded that the observed highly repeatable resistive switching between LRS and HRS (with a ratio of 6 × 10^2^) occurs due to the formation of conducting paths in the (Ti–Cu)Ox thin film. The results presented proved that thin films with a gradient element distribution profile can be an interesting configuration for the preparation of devices utilizing memory effects.
